# The Effects of an Individualized Smartphone-Based Exercise Program on Self-defined Motor Tasks in Parkinson Disease: Pilot Interventional Study

**DOI:** 10.2196/38994

**Published:** 2022-11-15

**Authors:** Heiko Gaßner, Jana Friedrich, Alisa Masuch, Jelena Jukic, Sabine Stallforth, Martin Regensburger, Franz Marxreiter, Jürgen Winkler, Jochen Klucken

**Affiliations:** 1 Department of Molecular Neurology University Hospital Erlangen Erlangen Germany; 2 Digital Health Systems, Fraunhofer Institute for Integrated Circuits (IIS) Erlangen Germany; 3 Medical Valley Digital Health Application Center GmbH Bamberg Germany; 4 Digital Medicine Group Luxembourg Centre for Systems Biomedicine (LCSB) University of Luxembourg Esch-sur-Alzette Luxembourg; 5 Digital Medicine Group Department of Precision Health Luxembourg Institute of Health (LIH) Strassen Luxembourg; 6 Digital Medicine Group Centre Hospitalier de Luxembourg (CHL) Luxembourg Luxembourg

**Keywords:** Parkinson disease, exercise, telemedicine, wearable sensors, patient-defined outcome measure, mobile phone

## Abstract

**Background:**

Bradykinesia and rigidity are prototypical motor impairments of Parkinson disease (PD) highly influencing everyday life. Exercise training is an effective treatment alternative for motor symptoms, complementing dopaminergic medication. High frequency training is necessary to yield clinically relevant improvements. Exercise programs need to be tailored to individual symptoms and integrated in patients’ everyday life. Due to the COVID-19 pandemic, exercise groups in outpatient setting were largely reduced. Developing remotely supervised solutions is therefore of significant importance.

**Objective:**

This pilot study aimed to evaluate the feasibility of a digital, home-based, high-frequency exercise program for patients with PD.

**Methods:**

In this pilot interventional study, patients diagnosed with PD received 4 weeks of personalized exercise at home using a smartphone app, remotely supervised by specialized therapists. Exercises were chosen based on the patient-defined motor impairment and depending on the patients’ individual capacity (therapists defined 3-5 short training sequences for each participant). In a first education session, the tailored exercise program was explained and demonstrated to each participant and they were thoroughly introduced to the smartphone app. Intervention effects were evaluated using the Unified Parkinson Disease Rating Scale, part III; standardized sensor-based gait analysis; Timed Up and Go Test; 2-minute walk test; quality of life assessed by the Parkinson Disease Questionnaire; and patient-defined motor tasks of daily living. Usability of the smartphone app was assessed by the System Usability Scale. All participants gave written informed consent before initiation of the study.

**Results:**

In total, 15 individuals with PD completed the intervention phase without any withdrawals or dropouts. The System Usability Scale reached an average score of 72.2 (SD 6.5) indicating good usability of the smartphone app. Patient-defined motor tasks of daily living significantly improved by 40% on average in 87% (13/15) of the patients. There was no significant impact on the quality of life as assessed by the Parkinson Disease Questionnaire (but the subsections regarding mobility and social support improved by 14% from 25 to 21 and 19% from 15 to 13, respectively). Motor symptoms rated by Unified Parkinson Disease Rating Scale, part III, did not improve significantly but a descriptive improvement of 14% from 18 to 16 could be observed. Clinically relevant changes in Timed Up and Go test, 2-minute walk test, and sensor-based gait parameters or functional gait tests were not observed.

**Conclusions:**

This pilot interventional study presented that a tailored, digital, home-based, and high-frequency exercise program over 4 weeks was feasible and improved patient-defined motor activities of daily life based on a self-developed patient-defined impairment score indicating that digital exercise concepts may have the potential to beneficially impact motor symptoms of daily living. Future studies should investigate sustainability effects in controlled study designs conducted over a longer period.

## Introduction

Motor impairment in everyday life is highly affected by prototypical symptoms of Parkinson disease (PD) including bradykinesia and rigidity reflected by slow and reduced amplitude of movements and limited automaticity [1]. Exercise and physical therapy are increasingly recognized as both effective and complementary—to dopaminergic medication—treatment of motor symptoms in PD [2].

### High-Frequency and Home-Based Training

There is evidence that high-frequency training (approximately 4 times per week) is necessary to gain lasting improvement in motor symptoms in individuals with PD [3,4]. This is a major limitation for typical outpatient settings owing to the high organizational burden for patients and therapists. Furthermore, quarantine-related isolation and canceled physical therapy sessions, owing to the global COVID-19 pandemic, have made a huge impact on patients with chronic diseases [5-8]. Therefore, new solutions in the form of remotely supervised, home-based exercise programs have been developed, and the COVID-19 pandemic has accelerated the acceptance of these telemedical solutions [8,9]. Several studies have shown evidence that prescribed home-based exercise improved motor symptoms in patients with PD [4,10-15].

Previous studies assessing the benefit of home-based exercise have included mild to moderate disease stages of PD (Hoehn and Yahr stages I-III) with no cognitive impairment, stable medication, and an average age >60 years [10-12,14-17]. Exercises performed (treadmill walking [16], cycling [12], and balance training [10,13]) as well as the type of presentation (personal visit, paper sheet, smartphone app, and instructional DVD) varied. All studies were, to a certain degree, supervised by a therapist and exercises were prescribed by a specialized therapist beforehand. The duration of intervention ranged between 4 weeks and 6 months [10-12,14-17]. Overall, these studies observed improvements by exercise programs in gait and mobility [4,10,14,15]. Studies that assessed the Unified Parkinson Disease Rating Scale, part III (UPDRS-III) score reported fewer motor symptoms after the intervention period, especially when compared with a control group without intervention [13,18]. One study reported an increased quality of life (QoL) [16].

A meta-analysis, including studies mentioned previously, showed that a minimum training frequency of 150 minutes per week for at least 6 weeks yielded considerably higher benefits than a lower training frequency. Furthermore, it reported a lack of sustainable effects, stating that benefits only lasted shortly after the end of the intervention [4], consequently confirming that a sustainable long-term solution is undeniably needed.

### Telehealth Solutions and Smartphone-Based Exercise

Exercise needs to be consistent, less supervised, and more personalized to reach full potential and become a sustainable long-term solution [4,19]. Telemedicine and new digital patient-centered technologies seem to be a promising solution to those problems, making exercise and feedback data more accessible [20,21]. We therefore developed a smartphone app to enable the home-based exercise program for patients with PD. Apps can provide the user with easy access and allow for remote supervision. As the content of an app can be modified rather easily, it allows for more personalization and an exercise program tailored to the individual requirements of patients.

Recent research showed that most patients with PD have access to mobile phones and internet and are comfortable with using these technologies [22]. Smartphone apps have already successfully been used in various studies within patients with PD [23-25]. One study in particular should be highlighted [12]. This study included 130 patients (Hoehn and Yahr stages under II) mildly affected by PD, aged 30 to 75 years with stable dopaminergic medication, being split into an intervention group performing 30 to 45 minutes of cycling on a virtual reality–enhanced home trainer at least 3 times a week for 6 months and a control group doing stretching exercises at the same frequency [12]. All participants received coaching instructions at the beginning of the intervention and a follow-up phone call every fortnight. The intervention included a motivational app that provided training instructions and tips, gave instant feedback, and monitored progress [12]. Outcomes were significantly lower UPDRS-III scores and an improvement in physical fitness; however, the benefits were only present during medical “off” state testing.

### Patient-Reported Outcome Measures

Patient-reported outcome measures (PROMs) describe how patients individually perceive the outcome of measures such as an intervention on their symptoms, functional status, or QoL [26,27]. As exercise programs aim to complement pharmacological therapies and to improve motor performance of patients with PD in everyday life, PROMs are of huge interest as they can be used as a measure of relevant intervention effects for individuals. Patient-defined outcomes are even more relevant in a patient-centered approach.

Despite the fact that the engagement of patients and the additive value of patient-reported outcomes has rapidly gained relevance, there is a lack of home-based exercise studies focusing on the individualized approach. This approach in particular might benefit substantially from using a modifiable smartphone app.

Therefore, the aim of this pilot study was to investigate the feasibility of a home-based, high-frequency exercise program for patients with PD in Germany, as a smartphone app–supported training program tailored to the individual patient requirements. In addition, we exploratory investigated possible improvements of motor symptoms associated with participating in the exercise program. Therefore, we included a structured evaluation of patient-defined outcomes such as individually relevant motor activities of daily living. These results need to be interpreted cautiously owing to the pilot design of this study but will serve as a valuable starting point for future high-quality hypotheses testing studies of our exercise program.

## Methods

### Study Design and Cohort

In this pilot interventional study, we focused on the feasibility of the digital intervention program. Individuals diagnosed with PD as defined by the Guidelines of the German Association for Neurology similar to the United Kingdom PD Society Brain Bank criteria [28] were included. They were recruited from regular visits at the Movement Disorder Outpatient Unit at the Department of Molecular Neurology, University Hospital Erlangen, Germany, in the time frame between March 2020 and October 2020. Patients aged >18 years with a Hoehn and Yahr disease stage between I and III were included. In addition to meeting the inclusion criteria, participants were required to use a smartphone to use the digital training app. Patients who reported motor fluctuations or dyskinesia were excluded. Patients continued their normal medication, exercise, and therapy throughout the study.

### Ethics Approval

This study was approved by the local ethics committee (reference number: 72_20 B, Medical Faculty, FAU Erlangen-Nürnberg, Germany) and participants gave written informed consent. This study was conducted in accordance with the Declaration of Helsinki.

### Assessments

Outcomes of the study were the scores or values of the outcomes of the parameters for System Usability Scale (SUS), Parkinson Disease Questionnaire (PDQ-39), patient-defined motor symptoms in everyday life, UPDRS-III, Timed Up and Go (TUG) test, 2-minute walking test, and sensor-based gait analysis. Each of these outcomes are explained in detail in the following sections.

The SUS was used to evaluate the usability of the app, in particular, if the app provides a clear and easy-to-use structure, if the system is consistent, and if participants feel comfortable using the app [29]. Usability is considered as good as indicated by a total score >68 [30]. PROMs consisted of patients’ self-perceived QoL (PDQ-39) [31] as well as patients’ reports on their personal motor symptoms and limitations to everyday tasks—defined by patients with support of a therapist. These motor tasks were documented on a scale between 0 and 10, where 0 represented no restrictions and 10 represented maximal restrictions in everyday life. Clinical assessments included the UPDRS-III [32] and the Montreal Cognitive Assessment [33].

Furthermore, sensor-based gait analysis was conducted including a standardized 4×10-m walk test, TUG, and a 2-minute walking test [34]. We used 2 wearable SHIMMER2 sensors (Shimmer Research Ltd) that were attached to the outer rear side of each shoe. Sensor signals were recorded within a (triaxial) accelerometer range of –6 to +6 g (sensitivity 300 mV/g), a gyroscope range of –500 to +500 degrees per second (sensitivity 2 mV/degree/sec), and a sampling rate of 102.4 Hz. The sensors were connected to a tablet via Bluetooth and the data were stored in the tablet [35]. A machine learning algorithm processed the stored data and calculated clinically relevant spatiotemporal gait parameters such as stride length and gait velocity [36,37]. This system has been proven to be technically valid [38]. More details on sensor-based gait analysis were presented in previous work [35-37,39,40].

### Development of a Smartphone-Based Exercise Program at Home

For this study, an interdisciplinary team of movement scientists, therapists, clinicians, and patients with PD developed an individualized and remotely supervised exercise program that was configured in the medical product smartphone app “PatientConcept” developed by NeuroSys GmbH, Germany. Data safety was based on the General Data Protection Regulation guidelines. The app provided digitally instructed personalized training videos to support self-sufficient training at home over a period of 4 weeks. At baseline, patients with PD reported between 4 and 7 individual motor impairments that hindered them in everyday life tasks (eg, “I have problems closing the buttons of my shirt. I would like to improve on that.”). On the basis of their impairment, PD-specialized therapists identified suitable training tasks. The individualized physical activity tasks were selected from eight categories (flexibility, strength training, gait, balance and posture, coordination and rhythm, large amplitude movements, finger and hand movements, and stretching). In total, 50 movement tasks in different levels of difficulty were available (eg, finger tapping improves fine finger motor skills that are needed for tasks such as closing buttons). Therapists defined 3 to 5 short training sequences, depending on the patients’ individual capacity, that were then configured in the patients’ app. In total, participants performed approximately 20 minutes of daily exercise training in their home environment using these 3 to 5 video sequences from the 8 categories. In general, approximately 15 repetitions per task (and if left-right-dependent per side) were required for each training session. For example, trunk stretching combined with arms lifting in a maximal stretched standing position was performed 15 times without using additional devices.

### Patient Education and Remote Support

**Figure 1:**
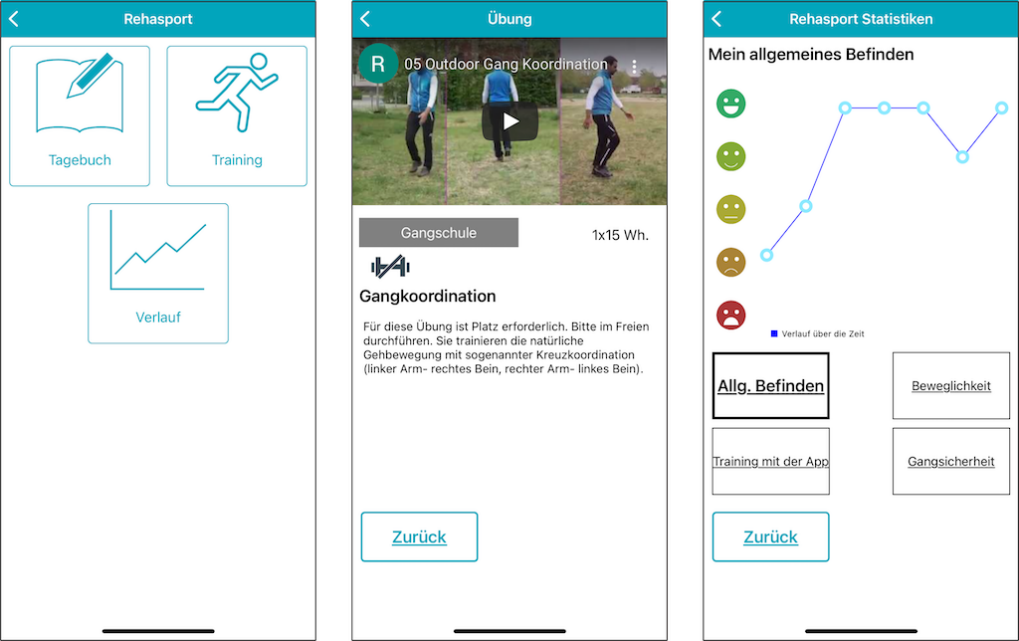
App interface (in German): home screen with a personal diary where participants gave feedback, the exercise button with personalized training videos, and a documentation sheet of the training progress (left); page for each exercise is presented—an instructional video, a short description, and the individually adjustable number of exercise runs (middle); and personal diary directly transmitted to the therapist (right).

In an initial education session for each patient at the University Hospital Erlangen, the training sequences were explained and demonstrated by a therapist. Patients were also thoroughly introduced to the smartphone app. Furthermore, to support the communication between the patient and (remote) therapist, we implemented a diary to document self-perceived general condition, mood, gait stability, and management of training sessions into the app. Using the diary as an interface, the therapist was able to directly supervise the progress of the patients. The app registered patients’ viewing of an exercise video and was thus able to monitor participation and adherence. Participants had the opportunity to directly contact their therapist via the smartphone app if they had questions or needed support. The smartphone app interface is illustrated in Figure 1.

Patients with PD were instructed to complete their short exercise program every day. At the midpoint of the study (14 days), a re-evaluation was scheduled. Depending on the preferences and training success of the patient, their schedule of exercise was updated, and some exercises were changed. At the end of the exercise intervention period, patients were asked to evaluate the applicability of the whole system including the smartphone app using the SUS.

### Statistical Analysis

Normality of data was tested by Shapiro-Wilk test and variance homogeneity by Levene test. As several parameters were not normally distributed, a conservative approach was used and nonparametric analysis was performed for all parameters. Repeated measures statistics (paired Wilcoxon test) was applied to analyze differences between baseline and follow-up visit. To minimize the effect of multiple comparisons among potentially related spatiotemporal gait parameters, significance level was adapted and *P* values of <.004 were considered as significantly different (*P*=.05/13; gait parameters=.004). For clinical assessments and PROMs, a significance level of *P*<.05 was used. All statistical analyses were performed using SPSS software package (version 24.0.0.2; IBM Corp).

## Results

### Study Cohort

Detailed information on the study participants is given in Table 1. In total, 15 participants completed the exercise sessions and final assessments. Adherence throughout the study was 100% as all participants completed the intervention program and performed their exercises reliably without any withdrawals or dropouts. All patients with PD (10 men and 5 women) were highly motivated to complete all training sessions within the time frame of 4 weeks. No adverse events were observed. The system usability score reached 72.2 (SD 6.5; 95% CI 68.5-75.8). Table 2 shows detailed results of each SUS question.

**Table 1:** Characteristics of patients with Parkinson disease at baseline (N=15).

Characteristics	Smartphone-based exercise at home	
	Mean (SD)	Range
Age (years)	66 (6.2)	55-79
Height (cm)	176 (7.9)	163-190
Weight (kg)	86 (17.7)	68-144
BMI (kg/m^2^)	28 (6.1)	21-46
Disease duration (years)	9 (5.7)	1-21
LEDD^a^ (mg/day)	566 (369.6)	105-1688
UPDRS-III^b^	18 (10.4)	4-43
MoCA^c^ (n=14)	28 (2.5)	22-30

^a^LEDD: levodopa equivalent daily dose.

^b^UPDRS-III: Unified Parkinson Disease Rating Scale, part III (motor score).

^c^MoCA: Montreal Cognitive Assessment.

**Table 2:** Overview of System Usability Scale (SUS) scores of all participants.

Patient	SUS 1	SUS 2	SUS 3	SUS 4	SUS 5	SUS 6	SUS 7	SUS 8	SUS 9	SUS 10	SUS score
Mean (SD)	4 (1)	2 (1)	4 (1)	2 (1)	4 (1)	2 (1)	4 (1)	1 (1)	4 (1)	2 (1)	72 (7)
#1	3	1	2	1	5	2	5	1	5	1	65
#2	5	1	5	1	5	1	2	1	5	1	67.5
#3	4	3	4	3	4	2	3	2	4	2	77.5
#4	3	1	2	1	1	4	3	3	4	1	57.5
#5	4	1	4	2	4	1	5	2	4	2	72.5
#6	4	2	5	1	4	1	4	1	4	1	67.5
#7	5	2	3	3	5	3	5	1	4	2	82.5
#8	4	2	4	1	4	3	5	1	4	2	75
#9	3	2	3	2	3	3	4	2	3	3	70
#10	5	3	5	1	4	2	5	1	5	1	80
#11	4	1	4	2	3	2	5	2	4	2	72.5
#12	5	1	5	1	5	1	2	1	5	1	67.5
#13	5	1	5	1	5	1	4	1	5	1	72.5
#14	5	1	5	2	5	1	5	1	5	1	77.5
#15	5	1	4	1	4	4	5	1	5	1	77.5

### Patient-Reported QoL

Participants completed the PDQ-39 questionnaire at baseline and follow-up to evaluate self-perceived QoL. The PDQ-39 total score as well as all subscores did not change significantly from baseline (mean 25, SD 12.7 points) to follow-up visit (mean 24, SD 13.3 points; *P*=.78). A descriptive improvement could be observed in mobility-related QoL by 14% (baseline: mean 25, SD 17.9 points; follow-up: mean 21, SD 16.9 points) and QoL regarding social support by 19% (baseline: mean 15, SD 16.5 points; follow-up: mean 13, SD 14.5 points).

QoL with regard to everyday life activities (washing, cutting food, or writing) and emotional well-being (feeling depressive or anxious) remained stable between baseline (mean 25, SD 13.5 points for everyday activities and mean 22, SD 13.0 points for emotional well-being) and follow-up (mean 26, SD 18.6 and mean 21, SD 16 points, respectively). Table 3 shows all data regarding QoL.

**Table 3:** Parkinson Disease Questionnaire (PDQ-39) scores for baseline (B) and posttest (P) subscores (N=15).

		SUS score		
		Mean (SD)	Range	Percentile, median (IQR)
**PDQ-39**				
	B	24.60 (12.72)	1-38	30.00 (13.00-35.00)
	P	24.07 (13.34)	3-49	28.00 (13.00-32.00)
**PDQ-39 mobility**				
	B	24.80 (17.93)	0-50	30.00 (5.00-42.00)
	P	21.27 (16.86)	2-55	22.00 (5.00-32.00)
**PDQ-39 everyday**				
	B	24.67 (13.49)	0-45	25.00 (16.00-33.00)
	P	26.40 (18.64)	0-66	25.00 (8.00-37.00))
**PDQ-39 emotion**				
	B	22.07 (12.98)	0-41	20.00 (12.00-33.00)
	P	20.60 (16.23)	0-50	25.00 (4.00-33.00)
**PDQ-39 SocialSupp**				
	B	15.40 (16.46)	0-41	8.00 (0.00-33.00)
	P	12.53 (14.54)	0-41	8.00 (0.00-25.00)

### Patient-Defined Impairment During Everyday Motor Tasks

**Figure 2:**
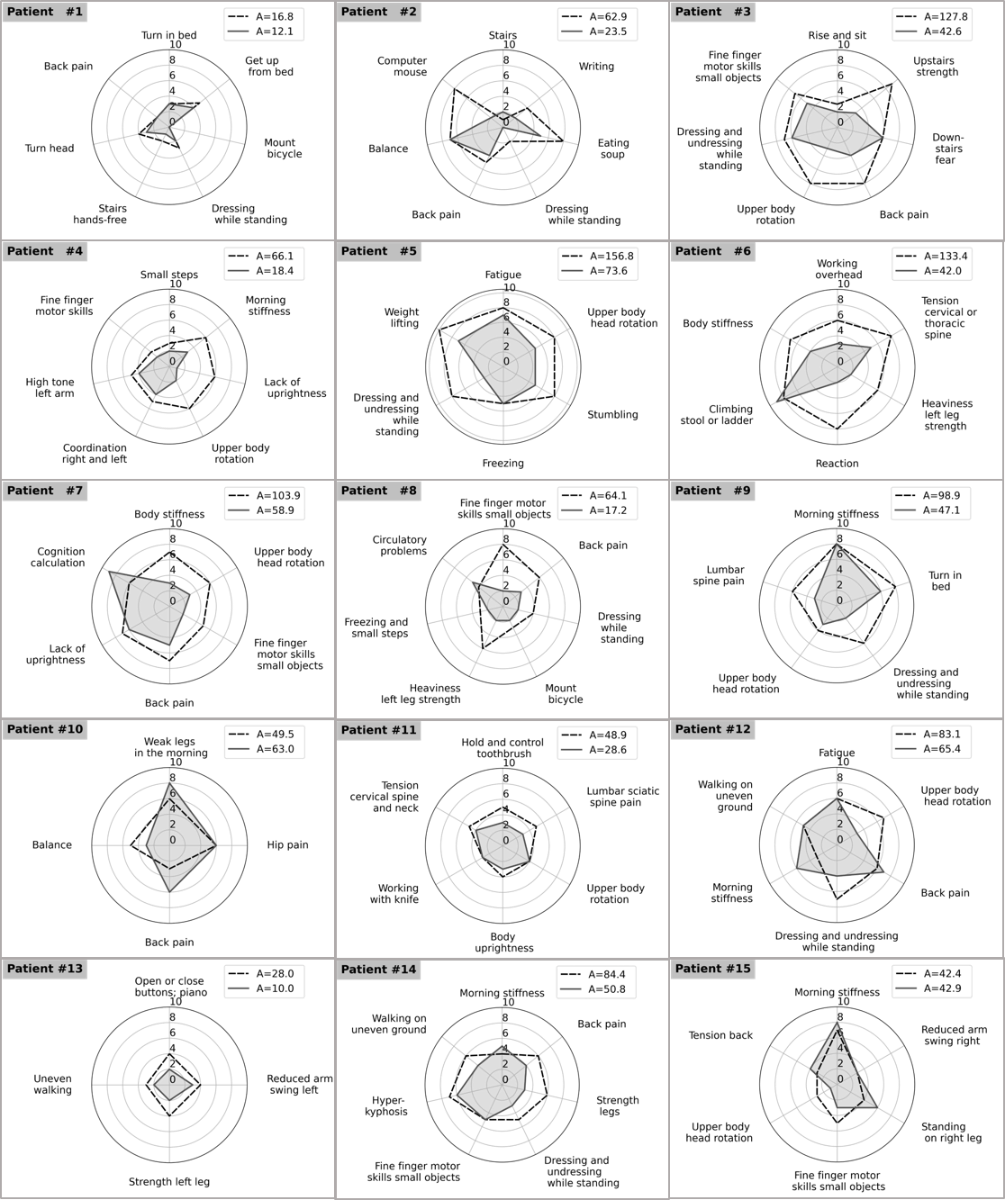
Radar plots of rated daily activity of all 15 patients. Dark dashed line represents Baseline, grey drawn through line represents Post Test.

Before the intervention, participants documented individual motor tasks in everyday life in which they recognized impairment (4-7 tasks were mentioned, such as limited trunk rotation, problems with buttoning up a shirt, or morning stiffness) and rated these deficits on a scale ranging from 0 (no impairment) to 10 (maximal impairment). After 4 weeks of daily personalized exercise, patients were asked to rate the same tasks again, without seeing their initial rating. Improvement or decrease in the activity was measured by comparing the areas outlined by the curves. The areas were calculated as consecutive triangles using the formula, 
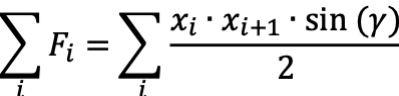
, wherein x represents the values stated by the patients, and the angle g is obtained by dividing 2π by i (the number of points measured). Overall, this resulted in a significant improvement by 38 (SD 31.5) units of area (approximately 40% on average; *P*<.001). Only one participant experienced an aggravation of 27% (13.5 units of area) owing to lower back pain that was unrelated to the intervention. Another patient remained stable. Figure 2 shows the individual everyday tasks each patient rated at baseline (dark dashed) and posttest period (grey drawn) as well as the resulting areas. As all patients additionally continued their normal therapy schedules during the intervention, a ceiling effect could be observed in two participants (participant #1 and #13).

### Clinical Motor Symptoms, TUG, 2-Minute Walk, and Gait Parameters

Motor impairment of patients with PD rated by a trained examiner using the UPDRS-III did not yield significant changes (*P*=.20) but descriptively decreased by 14% from 18 (SD 10.7) points at baseline to 16 (SD 6.7) points at follow-up. The conducted sensor-based gait analysis showed no change in TUG test, 2-minute walk test, or any of the measured gait parameters. A detailed comparison of these parameters between baseline and follow-up is presented in Table 4.

**Table 4:** Clinical scores and gait parameters at baseline (B) and posttest (P) period (N=15).

Parameters			Clinical score				
			Mean (SD)		Range		Percentile, median (IQR)
**UPDRS-III^a^**							
	B	18.13 (10.74)		4 to 43		19.00 (9.00 to 25.00)	
	P	15.60 (6.70)		6 to 29		16.00 (10.00 to 20.00)	
**TUG^b^ (s)**							
	B	11.05 (2.61)		7.8 to 16.2		10.10 (9.00 to 13.90)	
	P	10.08 (1.58)		7.6 to 13.1		9.55 (9.18 to 10.96)	
**2-minute walk (min)**							
	B	166.00 (31.88)		112 to 215		162.00 (150.00 to 190.00)	
	P	165.57 (24.38)		118 to 202		160.50 (149.75 to 191.00)	
**Stride time (s)**							
	B	1.09 (0.09)		0.96 to 1.25		1.08 (1.03 to 1.15)	
	P	1.09 (0.07)		1 to 1.24		1.08 (1.04 to 1.12)	
**Swing time (%)**							
	B	36.27 (1.61)		32.78 to 38.73		36.33 (35.60 to 37.61)	
	P	35.95 (1.75)		32.92 to 38.52		35.83 (34.49 to 37.65)	
**Strance time (%)**							
	B	63.73 (1.61)		61.27 to 67.22		62.67 (62.39 to 64.40)	
	P	64.05 (1.75)		61.48 to 37.08		64.17 (62.35 to 65.51)	
**Stride length (cm)**							
	B	133.19 (15.33)		108.68 to 157.5		135.31 (120.04 to 145.16)	
	P	132.67 (14.96)		106.24 to 158.9		135.00 (119.66 to 140.72)	
**Gait velocity (m/s)**							
	B	1.24 (0.18)		0.91 to 1.45		1.28 (1.06 to 1.41)	
	P	1.23 (0.17)		0.86 to 1.49		1.24 (1.12 to 1.36))	
**TO^c^ angle (degrees)**							
	B	−71.08 (8.10)		−83.12 to –56.24		−71.48 (−78.69 to −64.96)	
	P	−70.91 (7.72)		−82.56 to −60.44		−70.42 (−79.28 to −64.98)	
**HS^d^ angle (degrees)**							
	B	12.94 (7.93)		−5.24 to 31.42		13.07 (8.61 to 16.06)	
	P	14.11 (5.55)		1.86 to 27.42		14.15 (10.90 to 17.58)	
**Maximum toe clearance (cm)**							
	B	6.62 (2.04)		2.88 to 11.29		7.01 (5.58 to 7.70)	
	P	7.38 (2.04)		3.74 to 11.49		7.03 (6.45 to 8.50)	
**Stride time CV^e^ (%)**							
	B	4.07 (1.20)		2.09 to 6.4		4.08 (4.08 to 4.66)	
	P	4.10 (1.34)		1.84 to 6.84		3.99 (3.26 to 5.14)	
**Swing time CV (%)**							
	B	5.53 (2.13)		2.84 to 11.21		5.40 (5.40 to 6.26)	
	P	4.58 (1.33)		2.23 to 7.07		4.39 (4.39 to 5.56)	
**Strance time CV (%)**							
	B	3.15 (1.24)		1.41 to 6.54		3.09 (2.53 to 3.57)	
	P	2.61 (0.92)		1.23 to 4.39		2.47 (2.47 to 3.47)	
**Stride length CV (cm)**							
	B	6.82 (1.91)		5.08 to 12.3		6.32 (6.32 to 7.18)	
	P	5.89 (1.19)		4.06 to 8.28		6.08 (4.94 to 6.73)	
**Gait velocity CV (m/s)**							
	B	7.78 (1.93)		6.11 to 12.84		7.39 (6.21 to 8.10)	
	P	7.32 (1.62)		5.61 to 11.17		7.22 (7.2 to 8.40)	

^a^UPDRS-III: Unified Parkinson Disease Rating Scale, part III (motor score).

^b^TUG: Timed Up and Go.

^c^TO: toe-off.

^d^HS: heel strike.

^e^CV: coefficient of variance.

## Discussion

### Principal Findings

The aim of this pilot study was to investigate the feasibility of a home-based, high-frequency exercise program for patients with PD in Germany, as a smartphone app–supported training program tailored to the individual patient requirements. We exploratory investigated possible improvements of motor symptoms in a structured evaluation of patient-defined outcomes using individually relevant motor activities of daily living. The main finding of this study was that personalized home-based, high-frequency, digital exercise with remote supervision was feasible. In addition, the tailored exercise program was able to improve individual motor tasks regarding mobility and everyday life based on a self-developed patient-reported impairment score in this pilot study.

### Usability of App and Adherence

With an average SUS score of 72 (SD 6.5, 95% CI 68.5-75.8), the usability of the app used was considered good according to the commonly used averaged cutoff score of 68 [30]. In approximately 500 evaluation studies, the average SUS score was 68, implying that any score higher than that yields results above average. Even though there were a few technical difficulties, none resulted in dropouts or discontinuation of the training. We observed a very high adherence throughout the study. However, this high adherence has to be interpreted cautiously as the intervention period was limited to 4 weeks in this study. Whether this high adherence level is sustainable over a longer period, needs to be determined in future research. Similar apps for detection of speech impairment and sleep, motor, and emotional symptoms provide evidence that these digital tools beneficially complement the clinical diagnostics in patients with PD [41-43]. Consequently, digital health apps should be considered as a usable and relevant method in future research.

### PROMs: QoL Measures

Overall, we did not observe improvements in QoL measures (PDQ-39 total score). Previous studies presented contrary findings with regard to this aspect. Although some studies report increased QoL owing to exercise programs [16,44], others do not support the same [4,45]. There is evidence that motor symptoms rated by UPDRS-III and mobility have a smaller influence on QoL than nonmotor impairments such as mood or depression [46,47]. Exercise-related studies as this study or the ones mentioned in this paper mostly target on motor functions. Possible changes in QoL might therefore be overcovered by stronger influencing factors. However, when looking at the determined subscores of the PDQ-39 questionnaire, we observed a certain increase in mobility-related QoL, without reaching significance level in this pilot study. This underlines the improvements in self-defined motor tasks as previously described. Furthermore, there is a descriptive increase in social support–related QoL potentially indicating that patients felt supported by their therapists, even when solely digitally connected. QoL measures were already being implemented in several studies but have yet to improve care from a patients’ point of view [26]. Acknowledging the controversial results that have been reported with regard to QoL, further research is needed to understand the various aspects that influence QoL in patients with PD and which of them may be addressed by digital exercise programs.

### Patient-Defined Outcome Measures: Daily Motor Activities

A previous study investigated occupational therapy in patients with PD using a very similar PROM method and asked patients to list 3 to 5 daily tasks they aimed to improve and rated them on a scale from 1 to 10. Similar to our study, significant improvements in individually chosen motor tasks were observed while secondary outcomes such as UPDRS-III remained stable [48]. Another study has shown improvements in general self-reported mobility but did not specify the methods used [15]. PROMs gain in importance and priority as needs of patients with PD are being increasingly communicated and more recognized. For self-determination in patients with PD, the possibility to put emphasis on specific symptoms and decide which deficits they aim to focus on is very important for each individuals’ motivation [22]. Integrating patients and their needs into study designs is a crucial step to shape and develop a satisfying tailored approach [26]. As confirmed by these studies, by implementing their individual needs, patients with PD substantially benefit from exercise programs, even though clinical scores that were conventionally used to determine the effect of an intervention, such as the UPDRS-III, did not show significant improvements in this pilot study. PROMs may help managing and monitoring the progression of long-term medical conditions in PD [49]. Especially in the current change in health care and the rapid shift toward telemedicine that has been thriving throughout the COVID-19 pandemic, PROMs play a major role [9,27]. In summary, we highlight the importance of implementing PROMs (considered as patient-defined outcomes) into clinical studies and health care.

### UPDRS and Sensor-Based Gait Analysis

This study revealed descriptive improvements in UPDRS-III after a 4-week long home-based exercise intervention. We used a cutoff score of a 5-point difference in UPDRS-III between baseline and follow-up as the minimal clinically important change as is common for Hoehn and Yahr stages I to III [47]. Compared with other studies with comparable exercise interventions that yielded a significant difference in UPDRS-III, it is noticeable that these interventions lasted longer (8 weeks or 6 months) [12,13]. Considering that we observed a trend toward lower scores during follow-up testing in this pilot study, this suggests that the intervention period of our study was potentially too short for clinically relevant changes in UPDRS-III. However, our exercise program might be beneficial when conducted over a longer period. This theory is supported by a meta-analysis of different home-based exercise studies indicating that duration and frequency have a high impact on the outcome [4]. As our frequency was comparable with the suggested 150 minutes per week [4], a follow-up study with a longer intervention period might reveal potential improvements in UPDRS-III.

With regard to sensor-based gait parameters, a few studies observed improvements in some [10] or even on all aspects of gait (though the latter study used Nordic walking, focusing solely on gait and fitness and is therefore not directly comparable with our intervention) [44], when pooling different home-based studies, no significant long-term effect was observed [4]. This is in line with our findings and indicates that standardized gait tests in the hospital might not be the appropriate method to detect exercise intervention effects. Continuous home-based measures over a longer period may more precisely reflect the impact of therapy [50], as a broader picture of motor symptoms is drawn in comparison with snapshot measures on a certain time point of the day.

### Limitations

First, as this pilot study mainly focused on the feasibility of the intervention and the smartphone app, results were not yet compared with a control group. To fully evaluate the benefits of this intervention, future studies should include a control group matched for age, gender, and severity of PD-related symptoms. Consequently, this study was unblinded as assessors were aware that all participants received training. Second, owing to the low number of participants, the statistical power of the results of this study is rather low. Therefore, results presented in this study should be interpreted with caution. In this context, it should be considered that nonsignificant findings presented in the study may be either because of the actual absence of true effects of the intervention or because the statistical power was too low to detect true effects. Therefore, the results should be mainly considered as exploratory. Future studies with an active control group, randomized design, and blinded assessors should increase sample size and furthermore need to be conducted over a longer period to investigate whether this approach yields sustainable effects.

### Conclusions

In conclusion, this pilot study presented that an individualized, digital, home-based, and high-frequency exercise program over 4 weeks is feasible in patients with PD as indicated by a total SUS score of 72. The exercise program showed beneficial effects on individual patient-defined motor impairment in daily life activities (improvement of 40% on average). These results indicate that digital exercise concepts remotely supported by therapists have the potential to complement at-site exercise sessions and serve as additional stimuli in everyday life for patients with PD. This study also showed the relevance of a personalized exercise approach identified by individual, patient-defined outcomes. Future high-quality studies should investigate this digital intervention in more depth, evaluate potential gender-related effects, and whether clinically relevant effects are sustainable over longer periods.

## Abbreviations

PD: Parkinson disease
PDQ: Parkinson Disease Questionnaire
PROM: patient-reported outcome measure
QoL: quality of life
SUS: System Usability Scale
TUG: Timed Up and Go
UPDRS-III: Motor Score of the Unified Parkinson Disease Rating Scale, part III

## Data Availability

The data sets used and analyzed during this study are available from the corresponding authors on request.
